# P-485. Enhancing Adolescent Human Immunodeficiency Virus (HIV) Screening : A Quality Improvement Approach with Point-of-Care Testing

**DOI:** 10.1093/ofid/ofae631.684

**Published:** 2025-01-29

**Authors:** Mona Abutouk, Gabrielle Pina, Rachel Davidge

**Affiliations:** Loma Linda Children's Hospital, Loma Linda, California; Loma Linda Children's Hospital, Loma Linda, California; Loma Linda Children's Hospital, Loma Linda, California

## Abstract

**Background:**

Pediatricians play a key role in preventing Human Immunodeficiency Virus (HIV) by offering routine HIV testing. While there has been great progress in treatment for patients with HIV/AIDS, many adolescents still believe they are not at risk for HIV and have never been tested. Recent studies have shown that almost half of adolescents who have HIV are undiagnosed. A key barrier to screening adolescents for HIV during clinic well child visits includes patient follow through with HIV screening lab work. Traditionally, this requires a visit to the lab, serum draw, followed by waiting hours to days for a result. We sought to eliminate this barrier by implementing a point of care (POC) HIV oral swab during adolescent well child visits with anticipation of increasing overall HIV screening rates.

Plan Do-Study-Act Cycle

**Figure:**
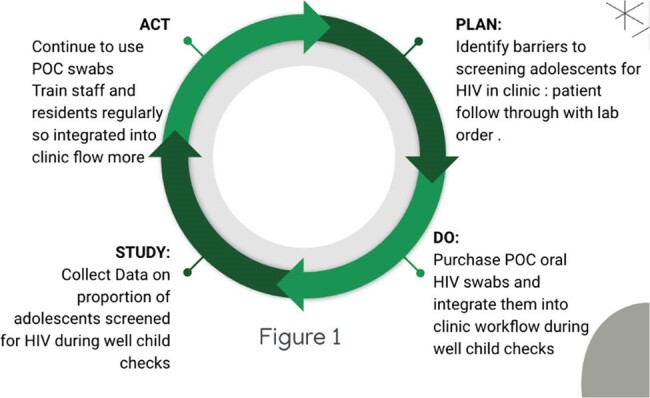

Multiple PDSA cycles were implemented throughout the project to ensure continued improvement and integration of HIV screening during clinic visits. This was the an example of the main overarching PDSA cycle driving quality improvement.

**Methods:**

Adolescents ages 15-18 presenting for well-child check who had not yet tested for HIV were eligible.

Interventions included creating educational curriculum for physicians regarding HIV screening guidelines, generating mulit-lingual resources for families about the importance of HIV screening, and utilizing a multidisciplinary team to implement testing into clinic workflow.

The primary outcome measure was the monthly percentage of adolescents ages 15-18 who were screened for HIV via the POC swab during their WCC.

Multiple “Plan-Do-Study-Act cycles” were implemented to reinforce change.

Proportion of Adolescents tested for HIV

**Figure:**
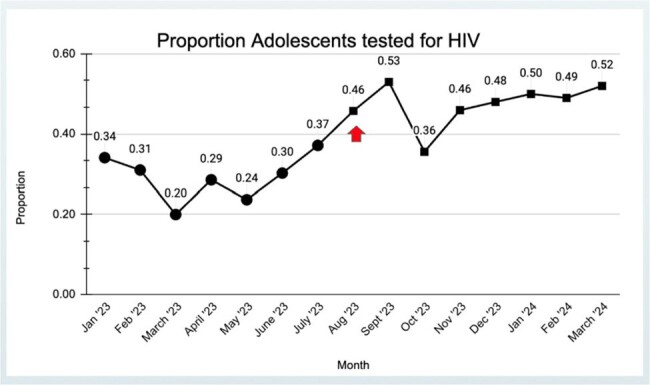

Red arrow indicated when POC HIV swabs became available for use in clinic

**Results:**

We found a statistically significant difference between the proportion of patients screened for HIV before and after the availability of POC HIV swab with p< 0.001.

Prior to implementation, the year-to-date percentage of those screened among eligible patients from January 2023 to July 2023 was 29% (80/276). Within eight months of implementation of the HIV POC swab from August 2023 to March 2024 the percentage of adolescents screened increased to 47 % (155/331).

**Conclusion:**

Improving HIV screening among adolescents is feasible and effective through the use of point of care rapid antibody testing. This type of testing is painless, can be quickly performed during clinic visits, and improves patient confidentiality as results are available at time of visit instead of traditionally waiting for the results by phone or email. Medicaid covers POC testing for HIV so this form of testing could be used in many clinics.

**Disclosures:**

**All Authors**: No reported disclosures

